# How Does SARS-CoV-2 Affect the Central Nervous System? A Working Hypothesis

**DOI:** 10.3389/fpsyt.2020.582345

**Published:** 2020-11-16

**Authors:** Fabio Panariello, Lorenzo Cellini, Maurizio Speciani, Diana De Ronchi, Anna Rita Atti

**Affiliations:** ^1^Department of Mental Health, Local Health Authorities, Bologna, Italy; ^2^Department of Biomedical and Neuromotor Sciences, Psychiatry, Bologna University, Bologna, Italy

**Keywords:** COVID-19, SARS-CoV, RAAS, ACE2, Ang(1-7)/Mas, brain aging, neurodegenerative and psychiatric disorders abstract, Alzheimer disease

## Abstract

Interstitial pneumonia was the first manifestation to be recognized as caused by severe acute respiratory syndrome coronavirus 2 (SARS-CoV-2); however, in just a few weeks, it became clear that the coronavirus disease-2019 (COVID-19) overrun tissues and more body organs than just the lungs, so much so that it could be considered a systemic pathology. Several studies reported the involvement of the conjunctiva, the gut, the heart and its pace, and vascular injuries such as thromboembolic complications and Kawasaki disease in children and toddlers were also described. More recently, it was reported that in a sample of 214 SARS-CoV-2 positive patients, 36.4% complained of neurological symptoms ranging from non-specific manifestations (dizziness, headache, and seizures), to more specific symptoms such hyposmia or hypogeusia, and stroke. Older individuals, especially males with comorbidities, appear to be at the highest risk of developing such severe complications related to the Central Nervous System (CNS) involvement. Neuropsychiatric manifestations in COVID-19 appear to develop in patients with and without pre-existing neurological disorders. Growing evidence suggests that SARS-CoV-2 binds to the human Angiotensin-Converting Enzyme 2 (ACE2) for the attachment and entrance inside host cells. By describing ACE2 and the whole Renin Angiotensin Aldosterone System (RAAS) we may better understand whether specific cell types may be affected by SARS-CoV-2 and whether their functioning can be disrupted in case of an infection. Since clear evidences of neurological interest have already been shown, by clarifying the topographical distribution and density of ACE2, we will be able to speculate how SARS-CoV-2 may affect the CNS and what is the pathogenetic mechanism by which it contributes to the specific clinical manifestations of the disease. Based on such evidences, we finally hypothesize the process of SARS-CoV-2 invasion of the CNS and provide a possible explanation for the onset or the exacerbation of some common neuropsychiatric disorders in the elderly including cognitive impairment and Alzheimer disease.

## Background

A novel respiratory illness was identified in Wuhan, the capital and the most populous city in the province of Hubei, in Central China in December 2019 (1–3). After an initial outbreak of infection at Huanan seafood market, possibly due to close animal-human contact, a new disease, now called coronavirus disease-19 (COVID-19) very quickly disseminated within China (4, 5). The novel coronavirus, called severe acute respiratory syndrome coronavirus 2 (and abbreviated SARS-CoV-2), is a positive-sense single-stranded RNA coronavirus coming from a bat coronavirus which spilled over to infecting humans after contaminating an intermediate host, maybe a pangolin (6, 7), which shares the genetic characteristics of the severe acute respiratory syndrome coronavirus (SARS-CoV) family with the 79% of RNA overlapping (as both SARS-CoV, the virus from which the 2002-2003 outbreak originated, and SARS-CoV-2 are classified among the beta-coronavirus phylogeny). This virus was firstly identified in patients and was hypothesized to be the etiopathological agent of the respiratory illness (1, 5). However, compared to SARS-CoV, SARS-CoV-2 appears to have significantly higher transmission capabilities which may be due to gain-of-function in binding to host cells. In the following months the infection was rapidly also detected in many countries outside China and just a month after the first identification of the virus, the World Health Organization (WHO) announced SARS-CoV-2 to be a “public health emergency of international concern,” and secondly a pandemic. By June 23rd, 2020, the pandemic had affected more than 200 countries, with 8,993,659 cases having been confirmed as COVID-19, including 469,587 deaths (8).

Growing evidence suggests that both SARS-CoV and SARS-CoV-2 appear to use the human angiotensin-convertase enzyme 2 (ACE2^1^) in order to infect host cells. With the aim to infect a host, the virus binds a molecule expressed by the cells of the latter (receptor) through its own protein that has the ability to bind it (ligand). The presence of the receptor allows the tissues that express it to become potential targets of the infection. Protein S is the main ligand that the SARS-CoV-2 virus uses to hook the ACE2 receptors expressed by the host cells and to infect their tissues. Protein S is divided into 2 subunits separated by a cleavage site (“furinic site”): the S1 subunit and the S2 subunit. The receptor expressed by host cells for SARS-CoV-2 S protein and SARS-CoV family viruses is the ACE2 protein. During the process of infection of the host cell, the S1 subunit binds to ACE2 and triggers a series of events that determine the process by which the S2 subunit determines the fusion between the viral capsid and the plasma membrane of the host cell. For this purpose, the action of the host protease transmembrane protease, serine 2 (TMPRSS2) that cuts the protein S in the 2 subunits at the level of the furinic site is necessary. This splitting process is essential to increase pathogenicity and improve the effectiveness of the merger process (13–15).

Since the beginning of the COVID-19 spread the most common clinical presentation of SARS-CoV-2 infection was characterized by mild to medium fever, dry cough, respiratory distress or dyspnea, with ground-glass pneumonia features on computed tomography (CT) scan (2, 16). Most recently, clinical reports were published demonstrating that SARS-CoV-2 affects the conjunctiva, the gastrointestinal tract, the heart and its pace, and may cause vascular injuries such as thromboembolic complications and Kawasaki disease in children and toddlers (17–19). A rapidly increasing number of evidences have also described neurological and psychiatric symptoms and complications, such as acute stroke (20, 21), hyposmia (22), Guillain–Barrè syndrome (23), and encephalitis (24). Emerging evidence suggests that the 36.4% of COVID-19 patients exhibit neurological symptoms including both central and peripheral signs (25). The first ones include consciousness-impairment, vomiting, headache, dizziness, and nausea, whilst the second ones are comprised of three types of hypoesthesia (hypoplasia, hypogeusia, and hyposmia), suggesting CNS-invading capabilities of the virus where it may affect the functioning of specific nuclei or neural circuits (25).

Among the neurological manifestations just described, those presenting early and those presenting later in the course of the COVID-19 pathology can be identified. Indeed agitation, confusion, and corticospinal tract signs affect above all patients hospitalized in intensive care units, COVID-19 can cause cerebrovascular ischemia and stroke also in young patients, Guillain-Barré syndrome (23), Miller-Fisher syndrome (26), and Kawasaki-like multi-system inflammatory syndromes now being recognized in children and teenagers by changing of coagulation and, in particular, to inflammation-induced disseminated intravascular coagulation (DIC) (20, 21). According to Heneka et al., it is possible to argue that four possible physiopathogenetic mechanisms through which SARS-CoV-2 affects the CNS can now be identified during the acute phase of COVID-19: (1) direct viral encephalitis, (2) systemic inflammation, (3) peripheral organ dysfunction (liver, kidney, lung), and (4) cerebrovascular changes. In the long term perspective, one or more of these mechanisms together may contribute to raise the risk for developing long-term neurological complications in COVID-19 survivor patients, either by worsening a pre-existing neurological disorder, or by onset of a new neurological pathology (19). This assumption is confirmed by the observation that about one third of COVID-19 patients discharged have cognitive and/or motor impairment (27). Connections between SARS-CoV-2 related infections and CNS pathologies are not to be unexpected, as the previously mentioned observations on COVID-19 appear to be in agreement with previous reports from Saudi Arabia in which significant neurological manifestations were found to be associated with Middle East Respiratory Syndrome (MERS-CoV) infection (28). Recent guidelines, however, do not include neuropsychiatric symptoms as typical COVID-19 symptomatology; for example, the WHO guidelines only report headache and altered mental status as neurological criteria for probable COVID-19 cases (29).

Older aged patients, especially males, and patients with medical comorbidities and frailty, appear to be at the highest risk of developing more severe clinical pictures, including neurological symptoms and a higher rate of systemic complications. Data from the National Survey of Residential Care Facilities in the United States showed that seven out of 10 individuals in assisted living had some cognitive impairment, ranging from mild (29%) to severe cognitive impairment (19%) (30). Not surprisingly, recent findings from Azarpazhooh et al. suggest a significant correlation between dementia, disability-adjusted life years (DALYs), and COVID-19 cases (31) with a rate of dementia in hospitalized cases ranging from 6.8% (32) to 13.1% (33). Moreover, dementia is a strong predictor of COVID-19 mortality (31) and raises the issue of how to safeguard and how to implement self-quarantine measures in these patients.

Given these evidences, the aims of the present speculative article are manifold: firstly, we will describe the pathophysiological mechanism through which SARS-CoV-2 infection causes COVID-19 in humans, and secondly, we will focus on literature data suggesting the mechanism through which SARS-CoV-2 hijacks the CNS. Lastly, our final main purpose, and the real innovative hypothesized theory, will be to describe the neuropathogenicity of SARS-CoV-2 with the aim to explain neurocognitive and psychiatric symptoms, which are based on pathophysiological data and scientific evidences adding our speculative pathogenetic theory to the four mechanisms proposed by Heneka et al. as described above. In more detail, we aimed to examine the role of consequences of ACE2 binding by SARS-CoV-2 in the CNS through the collection of evidence in preclinical and clinical studies outlining the subsequent increase and/or reduction of the main components of Renin Angiotensin Aldosterone System (RAAS) at the CNS level. Based on this evidence, we hypothesize a possible pathogenetic mechanism through which the brain and its functions can be clinically altered during SARS-CoV-2 infection.

## From SARS-CoV-2 to COVID-19: Pathophysiological Mechanism

The virus appears to be able to use two anatomical routes in order to reach, colonize and infect the CNS: (a) a body fluid pathway (such as liquor, lymph, or blood) and (b) a neural pathway. The main person-to-person routes of transmission for COVID-19 are close contact transmission and inhalation of respiratory droplets. Additionally, contact with the eye conjunctiva of SARS-CoV-2 containing droplets may allow, once the trigeminal nerve (V) is infected, for the virus to infect the brain by retrograde traveling. This route may result in impaired vision like hypoplasia. Additionally, SARS-CoV-2 can affect the sensory neurons which reach the taste buds of the tongue, from there it can infect the CNS through retrograde transport by reaching the nucleus of the solitary tract (VII, IX, and X) or the trigeminal nerve (V). This route may give a reason for hypogeusia. As the virus-containing respiratory droplets reach the mucous membrane that covers the nose, SARS-CoV-2 is also capable of entering the brain from the olfactory nerve (I), this may explain the clinical identification of hyposmia/anosmia in COVID-19 patients (34). In addition, in terms of body fluid invasion, the nasal mucosa provides a favorable environment for virus attack due to significant presence of blood and lymphatics capillary, which facilitate the entrance in the bloodstream after interaction with expressed ACE2 on endothelial cells. Finally, another modality of infection is the expression of ACE2 on epithelial cells that line the respiratory system, which enables respiratory viruses to cross into the bloodstream. The virus does not only use vascular pathways to spread into the CNS, neural pathways such as the vagus nerve branch (X) which innervates the respiratory system are used by the virus, causing clinical symptomatology such as dyspnea, dry cough, and worsening of acute respiratory distress syndrome (ARDS). Likewise, inadequate hand hygiene allows the virus to hijack the gastrointestinal tract and then to gain entry to the CNS through the blood vessels, lymphoid pathways, and the vagus nerve. Additionally, once the virus has entered the circulation it is also capable of invading the brain via the compromised blood-brain barrier (BBB), spreading to the liquor through leakage into the intracerebral lymphatic circulation of the CNS. Similarly, a damaged blood liquor barrier allows viruses in circulation to invade the fourth ventricle (34).

As upon described, recent studies confirmed that SARS-CoV-2 tethers to the ACE2 through their spike (S) protein (35, 36). Through the binding of the surface unit of the S protein (S1) to ACE2, viral attachment to target cells is facilitated. Additionally, once the receptor is bound, the virus has to access the cell cytosol in order to start its own replication, which is fulfilled by cellular serine protease TMPRSS2 through acid-dependent proteolytic cleavage of the S protein, a process similar to the priming of the S protein in SARS-CoV-2. After the binding between the S protein and ACE2, the S protein is then cut at both S1 and S2 sites level. This allows the exposure of the S2 site which allows the fusion of the viral and cell membranes. The step of cutting of the S protein through dibasic arginine sites by the protease TMPRSS2 that is expressed by the host cell to cleave the S protein in the S1 and S2 units is critical in order to allow both S2-induced membrane fusion and viral endocytosis with ACE2 in the host tissue (35, 36). A clathrin-dependent mechanism allows SARS-CoV-2 to be internalized, it then penetrates early endosomes. Once the spike protein of the virus comes in contact with ACE2 and binds it, the whole molecule or the transmembrane region of ACE2 enters the cell along with the virus by endocytosis. Subsequently, membrane fusion ensues and RNAs of the virus are released. The disintegrin and metalloprotease 17 (ADAM17) cuts the extracellular juxta-membrane region of ACE2. This phase is called “shedding.” In conclusion, the internalization and subsequent shedding of ACE2 diminishes the concentration of ACE2 itself on the surface of host cell (13).

As suggested by Wrapp et al. (37), the higher virulence of SARS-CoV-2 might be due to the higher affinity of the S1 protein for the ACE2 protein compared with that of SARS-CoV. The result of SARS-CoV-2-induced ACE2 internalization is the loss of expression of ACE2 at cell surface level, which would compromise the capability of the cell to metabolize Ang II, a key step for the cell to produce Ang-(1-7), which is one of the most important cardio-vascular mediators of the peripheral action of Renin Angiotensin Aldosterone System (RAAS). Therefore, the rise in the ratio of Ang II:Ang-(1-7) which follows ACE2 endocytosis may drive the damage to the tissue which is at first induced by SARS-CoV-2 infection. Thus, a diminished ACE2 expression at the cell surface level may contribute to chronic loss of affected tissues functions and, in our hypothesis, to generate brain-functioning impairment due to the neurotrophic properties of SARS-CoV-2 (13). Based on the collected evidence and these assumptions, we hypothesize that the reduced concentration of ACE2 and the consequent rise in the ratio of Ang II:Ang-(1-7) may be a causal factor in the genesis of the pathological involvement of the CNS and may participate in the genesis of neuropsychiatric symptoms and neurological clinical manifestations from COVID-19. Based on this evidence, we hypothesize a possible pathogenetic mechanism through which the brain and its functions can be clinically altered during SARS-CoV-2 infection, with a specific focus on impairment of cognitive function during and after COVID-19 and especially on the potential SARS-CoV-2-induced neurodegeneration.

## The Renin Angiotensin Aldosterone System (RAAS)

### Overview

Renin was the first component of the RAAS once it was discovered that extracts from rabbit kidney affected blood pressure (36, 38). Then it was found that the constriction of the renal artery led to high blood pressure, which drove to the discovery of angiotensin (Ang) (39, 40). Once Ang was purified, two forms were isolated and described: Ang I and Ang II. Thus, the existence of an enzyme capable of converting Ang into Ang I and Ang II was hypothesized. This enzyme, named ACE, was subsequently isolated and characterized by Skeggs et al. (41). An arm of the RAAS system which counterbalances the continuous production of Ang II was then described and characterized. Two independent research groups (42, 43) have thus isolated ACE2, which works to generate proteins with cardioprotective action. The human ACE2 (hACE2) is a zinc metallopeptidase comprised of 805 amino acids which shares 42% of the sequence of ACE in the metalloprotease catalytic regions, and it is able to cleave the decapeptide Ang I to Ang-(1-9) and to cleave the octapeptide Ang II to Angiotensin-(1-7) [Ang-(1-7)] (17). Ang-(1-7) seems to be the most relevant cardioprotective protein from ACE2 action. As Ang-(1-7) interacts with the Mas receptor (MasR), the Ang-(1-7)/MasR axis comprises the second arm of the RAAS axis (13), and it appears to have cardioprotective properties (44). Recently, some studies discovered the ACE2 protease domain to be the main receptor entailed in the onset of severe acute respiratory syndrome-coronavirus (45) and, more recently, as a receptor involved in the infection from SARS-CoV-2 (15, 46).

### Cascade

**Figure 1:**
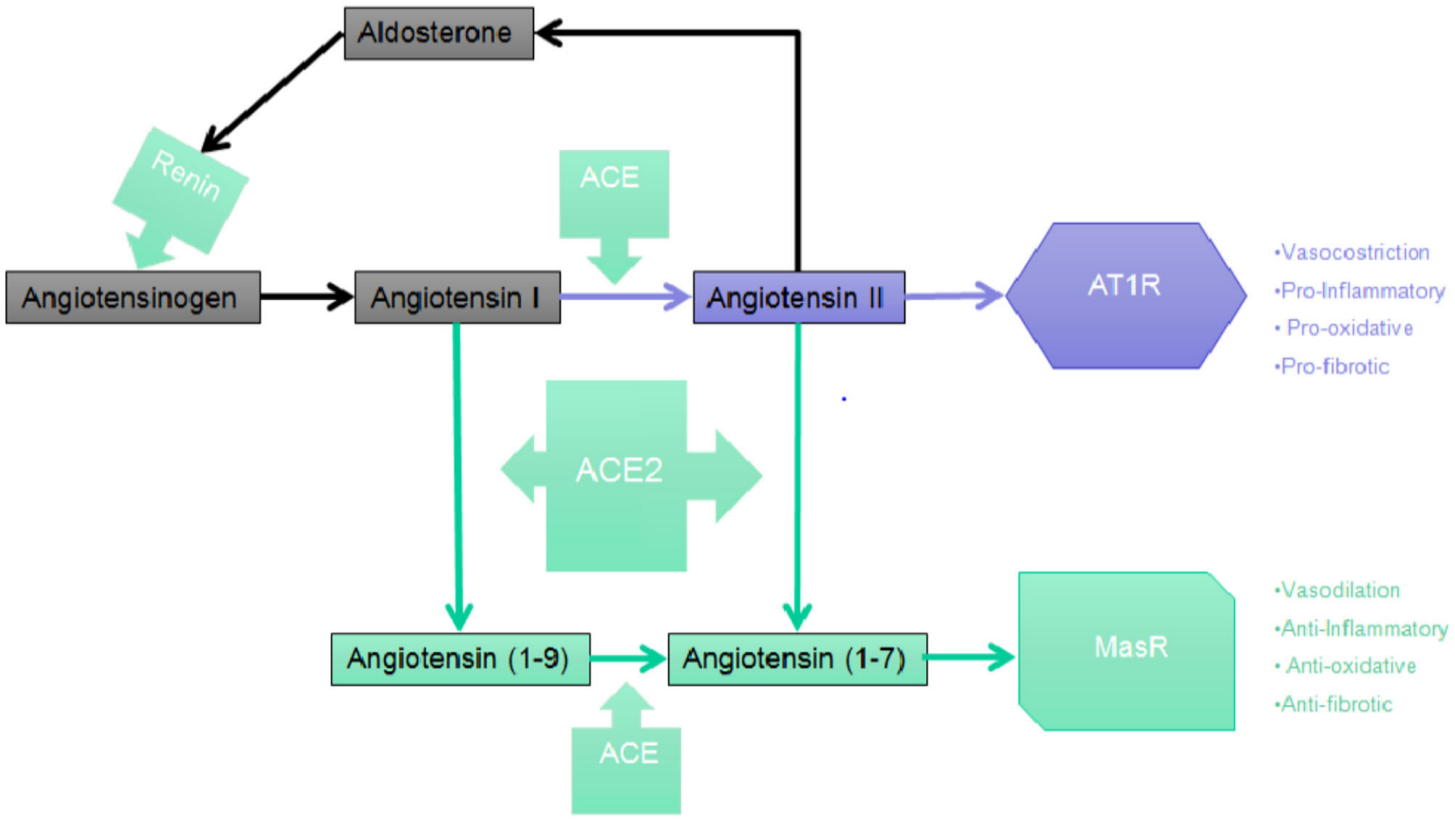
The RAAS cascade. Simplified picture of the Central RAAS pathway depicting the main steps leading to the synthesis of Angiotensin (1-7) which, in turn, binds and activates with the highest affinity the MasR.

The synthesis of renin by the juxtaglomerular cells (JG), which are located near the afferent (and sometimes also the efferent) arteriole of the glomerulus of the kidney, is the first step in the RAAS cascade. A precursor of renin in the form of a pre-pro-hormone is synthetized and it is then cleaved at its N-terminal of 43 amino acids, forming renin as an active compound. Renin is then stored in granules which are released into the renal and systemic circulation by an exocytic step involving coupling of stimulus-secretion (Figure 1). There are four interdependent factors which cause the secretion of the active form of renin: (1) alterations in the delivery of sodium chloride (NaCl) to the cells of the macula densa, which are located in the distal tubule and to the JG cells, together they constitute the “JG apparatus”; (2) changes of pressure in the perfusion of the kidney which are recognized by the baroreceptor mechanism in the afferent arteriole; (3) direct effect of Ang II on JG cells (negative feedback); (4) orthosympathetic stimulation through beta-1 adrenergic receptors (43). Renin, through the proteolytic removal of the N-terminus portion of angiotensinogen, is capable of regulating the first, rate-limiting step of the process in order to form Ang I, a biologically inert decapeptide.

The liver is the primary organ in which circulating angiotensinogen is synthetized, however mRNA expression of angiotensinogen has been identified in many other organs such as brain, kidney, vascular, placenta, adipose tissue, ovary, and adrenal gland. ACE then cleaves the C-terminal dipeptide of Ang I producing Ang II, a protein which, unlike Ang I, is biologically active and is capable of producing vasoconstrictor effects. ACE works also to metabolize many different peptides to their inactive forms, such as kallidin and bradykinin. Therefore, ACE effects may potentially decrease vasodilation and increase vasoconstriction (47, 48). Even though Ang II is the most known active product of the RAAS, studies suggest that different metabolites of both Ang I and Ang II may be capable of biological effects, especially in tissues. The sequential cleavage by aminopeptidases of amino acids from the N-terminal of Ang I and Ang II produces Ang III, a heptapeptide which is discovered in the CNS where it maintains tonic blood pressure and which play a role in hypertension and Ang IV, which derives from the subsequent enzymatic cleavage of Ang III (49). Ang II is converted by the action of carboxy- peptidases ACE2, that has a significant structural homology to ACE, to Ang-(1-7), an heptapeptide with biological activity. ACE2 has a role in the production of Ang-(1-9), another biologically active peptide from the cleavage of the C-terminal of Ang I.

### Angiotensin Receptors

Five subtypes of receptors mediating the effects of the RAAS biologically active peptides have been described as follows (49, 50):

- The type 1 receptor (AT1R), found typically in the form of a G protein-coupled receptor, which mediates the most well-known actions of Ang II, and among other functions it is involved in oxidative stress, inflammatory responses, and in the process of cell proliferation.
- The type 2 receptor (AT2R) is abundant during fetal life in the brain, kidney, and other sites, and its levels decrease markedly in the postnatal period.
- The type 3 receptor (AT3R) has unknown biological functions.
- The type 4 receptor (AT4R) plays a role in the mediation of Ang II, Ang III, and Ang IV in the release of plasminogen activator inhibitor 1.
- MasR is involved in vasodilatation, natriuresis, antiproliferation, heart protection, and brain function modulation. Such effects are due to the C terminal truncated peptide Ang (1-7) and not to the binding of Ang II.

### RAAS and CNS

Two different RAAS pathways have been described in the brain: the peripheral pathway, and the central pathway. The peripheral pathway allows for the peripheral access of RAAS components and involves both the forebrain and the circumventricular organs which surround the third and fourth ventricles, and it is constituted of fenestrated capillaries (51). Because of the BBB, which prevents peripheral RAAS constituents from entering most regions of the CNS, it is essential that there is synthesis of cerebral RAAS components in the brain. The central RAAS pathway is the main producer of locally synthesized angiotensin and links the medulla and the hypothalamus (51, 52). Additionally, other brain regions synthetize RAAS components as well. Both central and peripheral RAAS pathways contribute to the central control of cardiovascular homeostasis. In the CNS also, AT1R plays a role in vasoconstriction and is present on endothelial cells; on the contrary the AT2R plays a role in vasodilation (35, 51).

Ang II, Ang IV, Ang-(1-7), and Alamandine, that is produced from Ang-(1-7) via decarboxylase and from Angiotensin A via ACE2, are the main neuroactive forms of RAAS components. Ang-(1-7) binds to MasR with the strongest affinity, however it is also capable of binding AT2Rs and Mas-related-G protein coupled receptors (MrgDs). Alamandine attaches to MrgDs with the highest affinity. Ang II binds both AT1Rs and AT2Rs. Ang IV binds AT1Rs and AT4Rs. Receptors can be located on the plasma membrane of neuron, microglial cells and astrocytes, or intracellularly. The locations of intracellular receptors include neurosecretory vesicles, mitochondria and the nucleus. As previously described, ACE metabolizes Ang I into Ang II, and even though Ang II is capable of binding to both AT1R and AT2R, the upregulation of ACE increases AT1R activation specifically. AT1Rs are G-protein coupled receptors (GPCRs) which are located on basal ganglia, astrocytes, neurons, the hippocampus, microglia of the cortex and oligodendrocytes (53). The upregulation of ACE expression and the increase in the activation of AT1R signaling is a well-known process which regulates cell death, vasoconstriction (46, 47) and inflammation (15, 44). Conversely, AT2R, MasR, MrgD, and ACE2 possess vasodilation properties and are known for their positive effect on cognitive performance (50), promote the survival of cells (51), possess antioxidant effect (54), and promote anti-inflammatory processes (55). The MrgD, AT2R, and MasR pathways are interlinked, and reciprocally affect each other. MasRs and MrgDs ligands production is facilitated by ACE2. The activation of AT2R enhances ACE2 expression (36). A decrease in MasRs and ACE2 mRNA, protein and activity was found in knocked-out animal models (56). All these processes taken together appear to suggest a reciprocal interplay between enzymes and receptors in order to keep a balance in the maintenance of a well-functioning and healthy brain in terms of plasticity and resilience.

## ACE2, ANG-(1-7), and Central Nervous System: Evidences From Animal Models

The allocation of ACE2 in the CNS was under discussion since 2002 when suggestions of ACE2 mRNA were pointed out in the post-mortem human brain using quantitative real time polymerase chain reaction (RT-PCR) (57). Subsequently, with the aid of immunohistochemistry, ACE2 protein availability was found primarily at the level of endothelial and arterial smooth muscle of the vessel cells (16). Other evidence has outlined that ACE2 was found to be prevailing at the level of the glial cells (58). Additionally, Doobay et al. have outlined the presence of the mRNA and the ACE2 protein in the mouse brain, preponderantly in neurons (58). The evidence that SARS-CoV was found in infected patients brains, nearly always in neurons, substantiates the localization of ACE2 to the CNS (58, 59). Thanks to molecular biology techniques it has been found that ACE2 is ubiquitously spread throughout the brain, both in the nuclei that preside over the central modulation of cardiovascular functions (cardio-respiratory nuclei of the brainstem) and in brain areas responsible for other functions such as the motor cortex and the raphe (58).

While the role of ACE2 in the physiology and pathophysiology of the CNS is becoming better known, there is also an important body of knowledge supporting the fact that Ang-(1-7) plays a role in the brain. This peptide is mainly present in central brain areas linked to the control of blood pressure, such as the brainstem and the hypothalamus, and could play a synergic or opposite role on Ang II effects (60–63), as well as playing a role in neuromodulator action of cardiac baroreflex mechanisms and driving to a heightened responsiveness of this system (64), Ang-(1-7) has been outlined to roll out an relevant role in the negative modulation of norepinephrine release and to lead depressor responses in animal models, to enhance bradykinin levels, to boost the hypotensive upshots of bradykinin and to increase vasopressin and nitric oxide release (65–70). These effects are mediated by MasRs (65, 71), electively expressed in the CNS.

In spite of the fact that several data address that central ACE2 plays a predominant role in the conversion of Ang II into Ang-(1-7) in the brain, Elased et al. suggested that ACE2 activity in the CNS is more relevant than ACE activity under normal conditions compared to pathological conditions. This is completely inconsistent with previous findings proving that the physiological prominence of central Ang-(1-7) is uncovered in pathological circumstances and that its role is constrained in physiological conditions (72). On the other hand, it has been shown that the role in the CNS of ACE/Ang-(1-7)/MasR axis is not only limited to the control on cardiovascular function, but, in particular, thanks to the study of its inhibition, it has been highlighted that the ACE/Ang II/AT1R axis is involved in numerous other processes such as the regulation of the synthesis and release of neurotransmitters such as norepinephrine (NE), dopamine (DA), and y-aminobutyric acid (GABA) (72). For the sake of argument, in animal models Ang-(1-7) has proven to be capable to reduce the release of K+-induced NE in the hypothalamus, which in turn through a downregulation of the activity of tyrosine hydroxylase (TH) leads to a net reduction of the synthesis of NE. The fact that this inhibitory activity on NE release is experimentally blocked both by using a MasR antagonist such as A-779 and an AT2R antagonist such as PD123319, demonstrates the sharing of AT2R signaling in mediating this effect. Likewise, studies on aortic coarcted hypertensive rat models, show the ability of Ang-(1-7) to act inversely to Ang II on the release of hypothalamic NE, blocking its enhancing effects, and further showing the involvement of both receptor systems (MasR and AT2R). The administration of Ang-(1-7) to the rats in the striatum induces an increase in the release of both DA and GABA. The A-779, the MasR antagonist, is capable of inhibiting the increased release of GABA, but not of the DA; in order to obtain that result the co-administration of another antagonist is mandatory, EC33, which is an inhibitor of the enzyme that converts Ang-(1-7) in its metabolite Ang-(3-7). This evidence suggests that Ang-(1-7), through MasR, mediates the release of GABA, while the transformation in one of its active metabolites is fundamental to induce the release of DA (73).

### Central Cardiovascular Regulation

Evidence from animal models of hyper- or hypo-expression of ACE2 lead to the following findings.

The hyper-expression of ACE2 in the CNS is linked to a protective phenotype for the most common cardiovascular diseases (hypertension, chronic heart failure, cardiac hypertrophy). In fact, it entails a depletion of Ang II in the brain and consequently an enhancement in the amount of nitric oxide (NO), which would counterbalance and negatively modulate the peripheral cardiovascular effects of the Ang II mediated, instead, by the cutback of nitric oxide synthase (NOS) and sympathetic activity (74). Moreover, ACE2 hyper-expression in the brain mitigates the occurrence of deoxycorticosterone acetate (DOCA)-salt hypertension. Consistently, the low expression of ACE2 through experiments in transgenic animal model (mice) demonstrated a risen oxidative stress and autonomic response disruptions as opposed to controls. Starting from this evidence, Xia et al. hypothesized that the mechanism underlying the antihypertensive and autonomic disruption effect mixed up a switch in the balance between the central Ang II-AT1R and the Ang-(1-7)/MasR signaling in favor of the latter (75).

### Stroke and Brain Injury

Overexpression of ACE2 has been shown to mediate the circumscription of post-ischemic brain tissue damage in animal models (76–80) and, in particular, was combined with a lessening in the volume of the area of infarcted brain tissue under the same conditions (81, 82). The administration of the MasR antagonist, A779, was able to reverse these beneficial effects, suggesting once again how the pathophysiological mechanism underlying the extension of cerebrovascular damage following ischemia is recognized in the altered equilibrium between Ang II and Ang-(1-7) one of the main causal factors (76–80).

### Cognition and Memory

Recent evidence showed that Ang-(1-7) and its receptor MasR may be pivotal for memory handling in the hippocampus brain area (83). Congruently, *in vivo* studies with animal models of ACE2 hypo-expression demonstrated a worsening in memory and cognitive functions (84), and an intensified synthesis of reactive oxygen species and a simultaneous reduction in the production of the brain neurotrophic factor (BDNF). These changes reversed after the administration of AT1R and Ang-(1-7) antagonists, suggesting the important role played by the biochemical signal mediated by MasR in the positive modulation of these brain functions (83).

### Stress Response and Anxiety

Compared to controls, transgenic mice upregulating ACE2 exhibit behavior compatible with reduced anxiety levels (85). On the other hand, the MasR antagonist A779 reverts this behavior, suggesting that the Ang-(1-7)/MasR axis is involved in the modulation of anxiety levels and related behaviors. In a more recent study, using the same experimental model, Wang et al. reported a reduction in plasma corticosterone and proopiomelanocortin levels, assuming that ACE2 at the hypothalamic level by suppressing the synthesis of corticotropin releasing hormone (CRH) mediates the response to stress at the level of the hypothalamic-pituitary-adrenal (HPA) axis (86–89).

### Serotonin and Neurogenesis

A reduced synthesis of serotonin has been observed in genetically modified animal models for hypo-expressing ACE2 (90). Intriguingly, this reduction was correlated with the reduced intestinal absorption and consequently reduced plasma levels of its tryptophan amino acid precursor (91, 92). In fact, ACE2 has a non-catalytic role in the transport of amino acids (AA) in the intestine, and this notion has led to the hypothesis that the effects of ACE2 can be mediated, at least in part, by its actions on the gastrointestinal tract and/or on the intestinal microbiota. Among the multiple functions performed by serotonin in the literature, emphasis has recently been placed on neurogenesis. Indeed, Klempin et al. demonstrated that cell proliferation prompted by exercise in the dentate gyrus is abolished in ACE2-deficient mice. However, further studies will be needed to characterize the effective mediation of Ang II and Ang-(1-7), to confirm those pieces of evidence which are currently still contradictory (90, 93).

## ACE2, Neurological Functioning and Disease: Clinical Evidences From Preclinical Studies and Focus on Brain Aging and Alzheimer's Disease

The RAAS hyper-activation has been identified in several neuropsychiatric disorders, including Alzheimer's Disease (AD) and Mood Disorders (56). Since the lowest common denominators in all these pathologies are neurodegeneration, insulin resistance and the inflammation cascade, great attention has been paid in the literature to the possible relationships between the dysregulation between the two functional axes of the RAAS and the underlying neuropathological processes, since Ang II, as previously mentioned, is a pleiotropic factor locally metabolized in the brain (94).

Two critical studies show that Ang-(1-7)/MasR axis is chiefly involved in normal learning and memory processes. Among others, Hellner et al. outlined that Ang-(1-7)/MasR signaling augments long term potentiation in the CA1 region of the hippocampus, a key region for learning processes and implicit configuration memory (95). Correspondingly, Lazaroni et al. likewise demonstrated in an experimental animal model hindering MasR in the CA1 region of the hippocampus, object recognition memory was hampered (83).

Evidences accumulated over the years show the contribution of the RAAS components in the modulation of cognitive functions and an imbalance between the two functional axes of RAAS in both AD and mild cognitive impairment (96, 97). It's well-known that plasma renin and aldosterone levels decrease with advancing age (98, 99) although the underlying mechanisms are not fully understood and might include the age-related reduction in the number and in the functioning of nephrons and a reduced response capacity of RAAS to stimuli. First, the decrease in the number of nephrons induces a compensatory hyperfiltration by the remaining nephrons which determines an increase in the quantity of sodium chloride at the level of the macula densa with a reduction in the shaping and outflow of the renin and consequently in the synthesis of Ang II and aldosterone and therefore in plasmatic levels (99). Several studies on animal models of the aging process have shown that the decrease in plasma rates of Ang II is not parallel to that of renin. Few studies have been performed to evaluate Ang II levels in the elderly. For example, Duggan et al. showed a non-significant reduction in plasma levels of Ang II in a small sample of the elderly that did not include the so-called “older old” subjects (100). Second, in aged animals the release of renin in response to acute volume depletion or to sodium restriction is reduced compared to that of an adult animal. The tubular response to aldosterone administration is also impaired, as well as the response of plasma aldosterone to potassium infusion.

RAAS elements such as Ang II, Ang IV, and Ang-(1-7), and their receptors AT2R, AT4R, and MasR which positively affect cognition are abundant under physiological condition in many cell types such as neurons, astrocytes and microglial cells. Conversely, under pathological conditions such as post-stroke cognitive impairment (PSCI), vascular cognitive impairment (VCI), Parkinson's disease (PD), AD, or in the physiological aging process, the Ang II/AT1R axis prevails and cognitive functioning worsens (101).

*In vitro* studies show that the administration of Ang II blocks the K-dependent release of Acetylcholine in the temporal cortex (102), alters synaptic transmission in neurons of the lateral geniculate nucleus (103), and has shown, in *in vivo* studies, to suppress the induction of long term potentiation (LTP) in the lateral nucleus of the rodent amygdala (104). The cholinergic system at the central level is notoriously directly involved in cognitive, arousal and attention processes (105), LTP is considered to be a neuronal model of learning. The induction effect on it is probably mediated by the action of AT1R as it is reversible upon administration of the specific AT1R antagonist Losartan, while this does not occur after administration of the specific AT2R antagonist (PD123319) (104). *In vitro* studies show that Ang II influences as well-long term depression (LTD) in the lateral amygdala by means of a mechanism involving L-type calcium channels and AT1R, suggesting a role for the plasticity changes in the lateral nucleus and a possible cellular mechanism essential for the beneficial effects of ACE inhibiting drugs on the cognitive improvement in AD (96, 106).

*In vivo* studies on animal models have only partially confirmed the above suggested *in vitro* (107). Through behavioral analysis in different tasks after administration of losartan, PD123319, or both, it has been found that both receptors, AT1R and AT2R, are involved in memory enhancement processes, albeit with different power and intensity, showing a preferential involvement of AT2R in the enhancement of acquisition and recall of avoidance behavior (108). Other studies, on the other hand, using learning tasks have diminished the role of endogenous Ang II by suggesting that in CA1 it does not modulate memory consolidation through AT1R and AT2R (109, 110). Using a different experimental paradigm, Akhavan et al. shows that Ang II display an important role in brokerage of the effect of exercise on learning and memory, although the basic biochemical mechanisms remain largely unknown (111).

**Figure 2:**
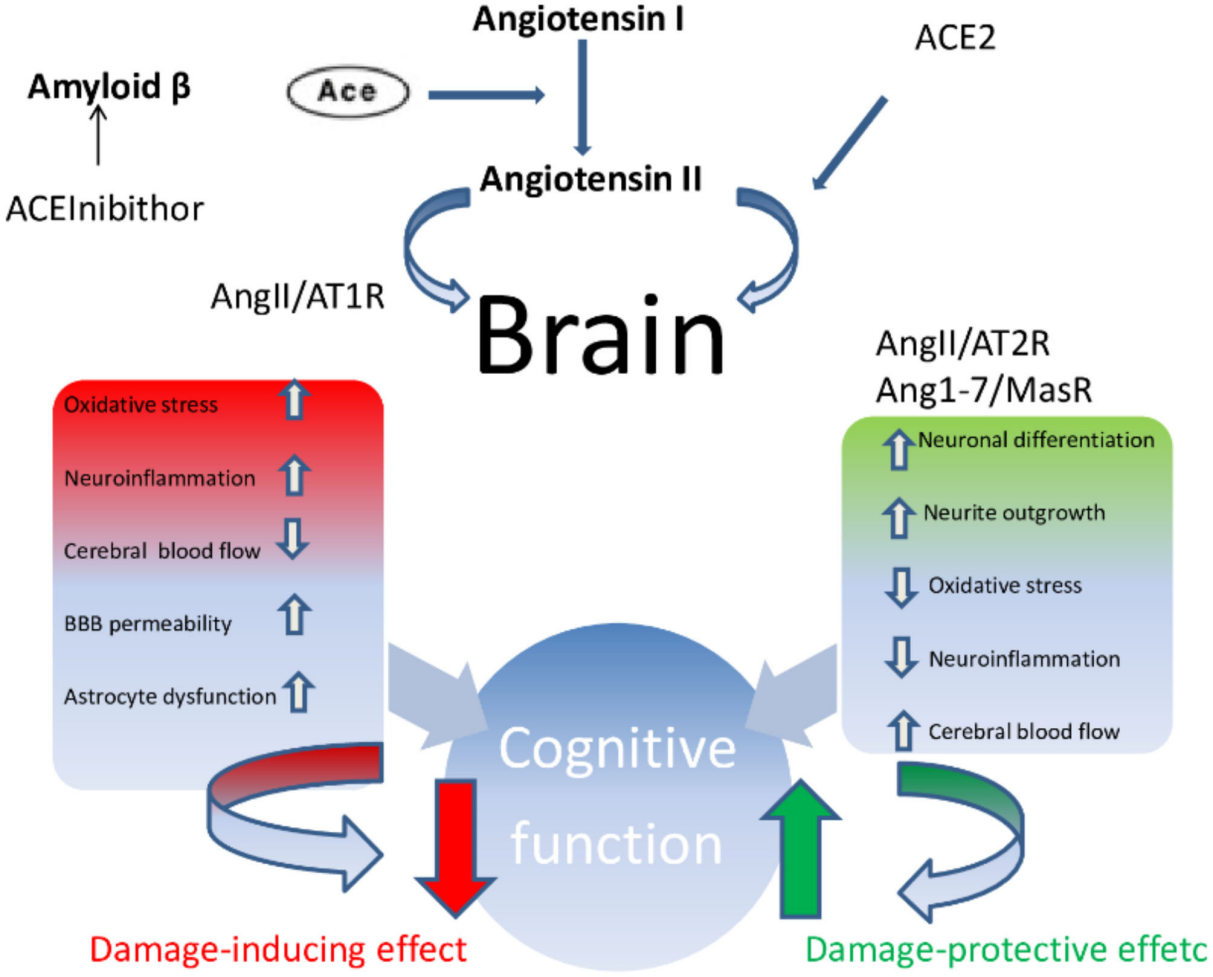
Ace/AngII/AT1R axis–Ace/Ang (1-7)/MasR axis imbalance. In condition such as AD, vascular cognitive impairment and post-stroke cognitive impairment, the Ace/AngII/AT1R axis predominates magnifying and accelerating the development of cognitive impairment.

**Figure 3:**
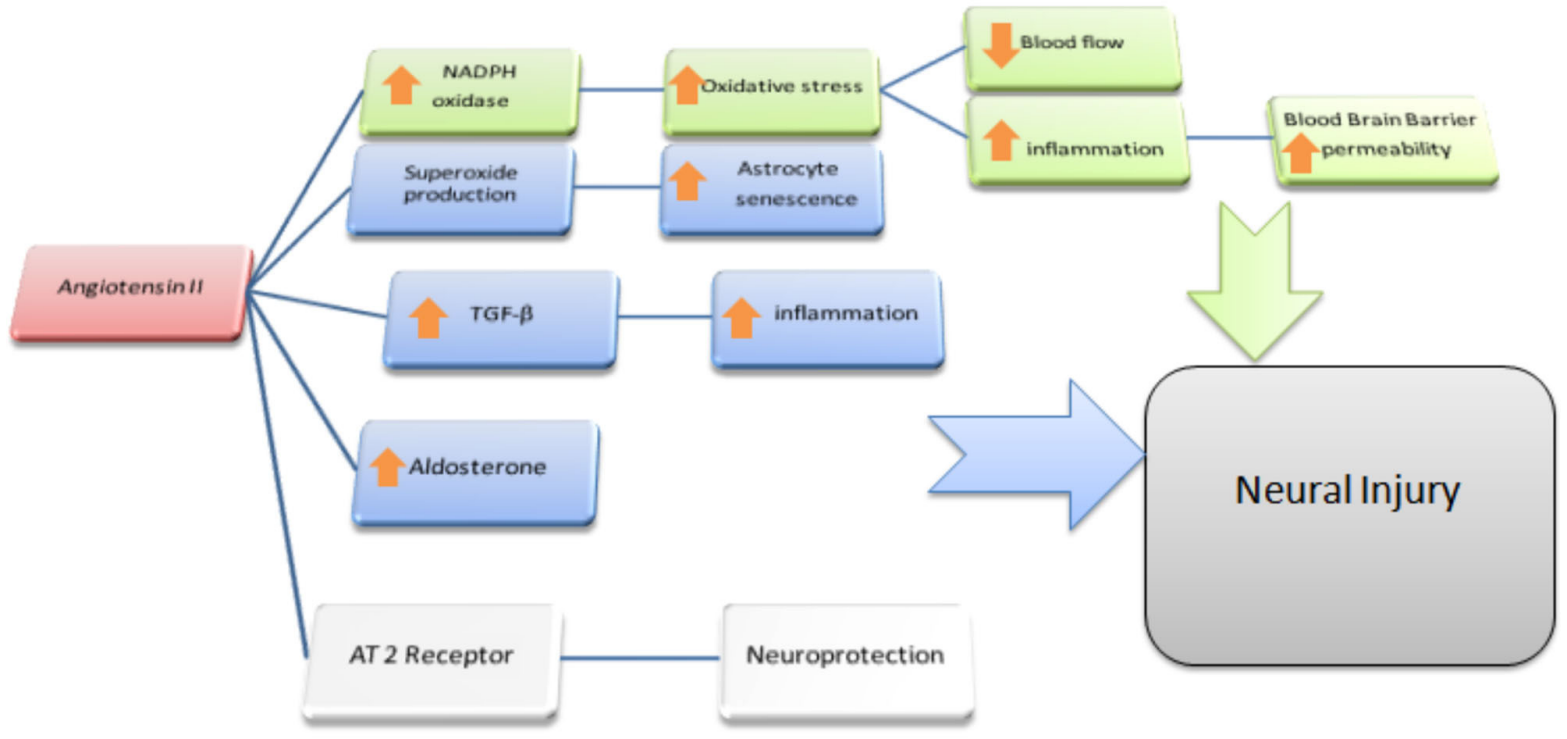
Ang II and the Brain. Possible effects of Ang II on Non-neural cells: In green is outlined the cascade responsible for the reduction of blood flow and enhanced permeability of the BBB at the microvessels level; we depict in blu the reported effects of Ang II acting as a paracrine mediator in CNS: mainly in astrocytes where, via TGF-β upregulation and local aldosterone production, it exerts pro-inflammatory effects resulting in indirect neural damage. Moreover, via superoxide production, Ang II accelerates senescence and dysfunction of astrocytes itself. Both these processes are supposed to be cofactor in leading to cognitive dysfunction resulting in higher susceptibility to dementia.

In summary, a growing body of scientific evidence pointed out that the upregulation of ACE2 and an increased proportion of Ang-(1-7)/Ang II, parallel to the positive tailoring of Ang II signaling through AT2R and Ang-(1-7) through MasR, determine an improvement in cognitive function and is involved in the treatment of dementia, above all AD (Figure 2). More specifically, the cognitive outcomes of Ang II deficiency and/or abundance (Figure 3) have been studied above all in preclinical model studies. Even though the BBB is impervious to all RAAS components, it was hypothesized that the local brain RAAS may possess pharmacological and physiological properties in the CNS (112). Inconsistent findings about the contribution of Ang II in memory and learning process *in vivo* studies were reviewed by Gard (113). Learning and memory in rodents were found to be enhanced by Ang II (114), however other studies found evidence of Ang II harming cognitive function (115). Experimental evidence reports that one possible reason is that the short-term effect of Ang II would consist in improving cognitive functions; on the other hand, Ang II in the long term could contribute to the functional exhaustion of neurons and consequent cognitive deterioration. That may be because of the induction of cerebrovascular remodeling by Ang II, which, by driving oxidative stress and vascular inflammation, produces an impairment in cerebral blood flow regulation (CBF) (116, 117). Additionally, endothelial capacity in brain vessels was affected by central expression of Ang II in genetically modified animal model of Ang II-dependent hypertension (118, 119). Moreover, Ang II was capable of inducing astrocyte senescence, a process involved, via superoxide production, in age-related neurodegenerative disease (120). Conversely, perindopril, which acts as a centrally active ACE inhibitor, was found to counter cognitive dysfunction in a mice model of AD and in chronic central hypoperfusion rats (121). These results suggest that permanent Ang II stimulation negatively affects cognitive function through the stimulation of the AT1R via degradation of neurons such as an increase in cellular senescence, CNS inflammation and oxidative stress, and through a decrease in the liquor in the brain. Cognitive impairment then follows neuronal degeneration, as induced by the many stimuli of Ang II. In terms of the clinical relevance of the RAAS cascade modulation and neurodegenerative disease, we will focus on the epidemiologically most impacting dementia: AD.

Two main pathophysiological mechanisms have been proposed to explain neurodegeneration as a pathogenetic mechanism involved in AD: (i) the hypothesis based on amyloid cascade s and (ii) the hypothesis based on cholinergic neurotransmission. According to the former hypothesis, neurodegenerative aberrations that bring to clinically relevant AD are induced by Aβ (1–42) (122). More specifically, the cleavage of the amyloid precursor protein (APP) produces a peptide, Amyloid β (Aβ), which is a 39–42 amino acid peptide (123) ACE appears to affect Aβ metabolism, thus suggesting a link between RAAS and AD (124). ACE contributes to degradation of β-amyloid in the brain, that is responsible for AD and ACE2 mediated release of Ang-(1-7) peptide in nerve tissue has potential neuroprotective actions. Taking together these findings outline that the smaller ratio of ACE/ACE2 score may contribute to the onset or the speeding process of pathophysiology of AD.

With regard to the cholinergic hypothesis, a depletion of neurons characterizes AD, in particular of those neurons which express nicotinic acetylcholine receptors (nAChR) (125, 126). Moreover, even though few studies investigated the link between Ang II and α7nAChR, Marrero et al. found that Ang II appears to activate the tyrosine phosphatase Src homology region 2 domain-containing phosphatase-1 (SHP-1), resulting in the block of neuroprotection against Aβ(1–42) mediated by nicotine (127, 128). Additionally, they found Ang II to be capable of inhibiting in PC12 cells, via SHP-1 activation induced by AT2R, the α7nAChR-induced activation of the JAK2-PI-3 K cascade (128, 129).

*In vivo* model evidences of the involvement of RAAS in the neurodegenerative disorders mainly come from genetic studies and cerebrospinal fluid (CSF) levels of the metabolites of the RAAS cascade. Significant single nucleotide polymorphisms (SNPs) in ACE gene also showed association with AD risk. The ACE gene insertion/deletion (I/D or indel) polymorphism has long been linked to AD. Fekih-Mrissa et al. have outlined that there was a significantly increased risk of AD in carriers of the D/D genotype (51.67% in patients vs. 31.67% in controls; *p* = 0.008, OR = 2.32). The D allele was also more frequently found in patients compared with controls (71.67 vs. 56.25%; *p* = 0.003, OR = 2.0). Moreover, as assessed by Mini-Mental State Examination, patients suffering from severe dementia were found predominantly in the D/D carriers group and, conversely, the D/D genotype and D allele were more frequently found in AD patients with severe dementia (130).

From a biochemical point of view, in 1986 Zubenko et al. showed that mean levels of the hydrolase ACE in CSF samples from a group of patients with dementia of the Alzheimer's type, were decreased (131). More recently, Kauwe et al. have conducted a genome-wide association study of CSF levels of 59 AD-related analytes. All analytes were measured using the Rules Based Medicine Human Discovery MAP Panel, which includes analytes relevant to several disease-related processes. They identified genetic associations with CSF levels of five proteins involved in amyloid processing and pro-inflammatory signaling. Among these proteins there was ACE, and SNPs associated with ACE protein levels are located within the coding regions of the corresponding structural gene. The genetic associations reported were new and suggested mechanisms for genetic control of CSF and plasma levels of these disease-related proteins. Significant SNPs in ACE showed association with AD risk in this study as well (132).

Taking together all these findings, it is possible to argue that the RAAS cascade is involved in neurodegenerative process. More specifically the constant activation by Ang II is capable of damaging neurons through AT1R stimulation via multiple cascades. Conversely, AT2R stimulation appears to protect against cognitive impairment, neural damage and the senescence process.

## RAAS and Psychiatric Disturbances

### Stress Related Disturbances

RAAS has been considered a stress response system similar to the HPA axis in which Ang II is considered an important stress hormone (133) that binds AT1R and AT2R located on stress-sensitive brain areas, including the HPA axis, amygdala, hippocampus and prefrontal cortex (134).

Similarly to the HPA system and its effects on cortisol, the RAAS cascade in humans has been considered a stress response system and higher levels of functioning are observed both following acute stress-related tasks and following stress chronically induced (133). Ang II is nowadays considered one of the most important stress hormones of the RAAS cascade, through the link with its AT1R and AT2R receptors in specific CNS regions such as the amygdala, the hippocampus, the prefrontal cortex and modulates the HPA axis, in particular through the link with paraventricular AT1R (133, 134). In fact, ACE inhibitors proved effective in regulating and desensitizing the HPA's response to stress (135). It is mandatory to mention that the effects mediated by AT2R in general counterbalance the action of Ang II on AT1R receptors, whose inhibition represents the main biological pathway of stress resistance and resilience (135).

Despite several clinical studies, there is still little evidence aimed at investigating the role of RAAS as an intervention target for the modulation of anxiety and stress response. In this regard, a 2012 observational study in patients suffering from post-traumatic stress disorder (PTSD), in which both the use of the AT1R antagonists and ACE inhibitors were associated with a protective profile regarding anxiety and fewer symptoms of the anxious spectrum in the patients examined (136). Unlike the RAAS cascade, ACE2/Ang-(1-7)/MasR axis has accumulated an increasing number of scientific evidences that qualify it as a protective factor in various neuropsychiatric pathologies, including psychosis, major depressive disorder (MDD), AD, PD, and stress disorders (79, 83, 88, 137). The protective effects on the central nervous tissue are mediated by anti-inflammatory and antithrombotic actions, as well as by the reduction of oxidative stress and apoptosis mediated by the latter (76, 138).

The central administration of Ang-(1-7) reduces the autonomic response to stress, reducing the high levels of stress-related hormones in the CNS, including Ang II itself, serotonin, DA and NE in the critical cerebral regions for this reply. MasR-KO mice in experiments showed increased durability of LTP and higher anxiety-like symptoms (87). The injection of Ang-(1-7), on the other hand, enhanced LTP through its action on NO and cyclooxygenase-2 in the lateral amygdala (139). Another study on the anxiolytic effects of Ang-(1-7) identified in the amygdala a correlation between the anxiolytic effects and the reduction of oxidative stress markers, and contextually the increase in the activity of glutathione peroxidase (140).

Consistent results were observed in transgenic mice overexpressing ACE2 and the GABAergic transmission: these mice tend to present an increased GABAergic tone specifically in the basolateral amygdala. High degree of ACE2 amount corresponds to high levels of Ang-(1-7) production which would induce an increased release of GABA locally, responsible for the anxiolytic effects observed (85). Another study found that in transgenic animal model (mice) with down-regulated synthesis of glial angiotensinogen, lower levels of serotonin synthesis and release in frontal and parietal cortex as well as in the hippocampus, which in turn could account for the depressive behavior shown by the experimental animals. Interestingly, this behavior is reversed both by the treatment with the serotonin selective reuptake inhibitor (SSRI) antidepressant fluoxetine, and by Ang-(1-7) injection (141).

### Affective Disorders

There is not enough experimental evidence to evaluate the potential contribution of the components of the RAAS cascade as a biomarker or as a target of treatment strategy for affective disorders. However, studies have shown that drug free and/or naïve patients with a first episode of MDD have significantly higher circulating plasma levels of RAAS cascade components than healthy controls. Among the various components of the RAAS cascade, attention was paid to circulating levels of Aldosterone as a promising biomarker of affective disorders: in a study, low aldosterone levels related both to a greater clinical severity of depression and to an increase in suicidal behaviors (142).

### Psychosis

In consideration of the aforementioned RAAS action on the modulation of the release and synthesis of DA (143), the potential role of ACE in the pathophysiology of schizophrenia and pathologies of the psychotic spectrum has been investigated. The results of the clinical studies carried out so far show contradictory results. In a recent study compared to healthy controls, patients with schizophrenic spectrum disorder show higher levels of circulating ACE (144–146). In contrast to these results, Wahlbeck et al. reported lower ACE activity than in controls examining the liquor of patients affected by schizophrenia (both in pharmacologically treated and in drug-free patients) (147). They also observed an inverse correlation between the enzyme activity of ACE and the CSF levels of DA and NE (148). In part, these conflicting results are attributable to methodological limits in the selection of the sample, since the population was not homogeneous regarding illness duration and drug co-treatment. Further studies that consider a greater stratification of the sample could shed light on the possible role of RAAS in disorders of the schizophrenic spectrum.

## Our Generating Hypothesis

Considering the experimental data exposed and the scientific evidence mentioned so far, the mechanism of the cascade of the RAAS axis is characterized by the dynamic balance of two arms with a mutually counter-regulatory function. The first arm is that composed of the ACE/Ang II/AT1R with proinflammatory activity and the second arm is composed of ACE2/Ang-(1-7)/MasR with anti-inflammatory properties (149). In this context, the binding and subsequent modulation of the expression of ACE2 by SARS-CoV-2 would therefore not only be the way through which the virus generates the infection but also one of the main pathophysiological mechanisms of COVID-19. The disease would develop at least in part as a consequence of the imbalance of this dynamic balance in favor of the hyperactivity of the ACE/Ang II/AT1R branch due to the reduction in the expression and activity of the ACE2 enzyme. In fact, ACE2, following the interaction with the SARS-CoV-2 protein S, would undergo a process of endocytosis mediated by membrane enzymes with consequent reduction of the transformation of Ang II causing Ang-(1-7) hyperstimulation of AT1R and a higher prevalence of proinflammatory activity with a subsequent storm of cytokines leading to tissue damage. Data consistent with this deduction come from *in vivo* studies with animal models (mice) of lung injury. In fact, in these models a reduced expression of ACE2 and an increase in Ang II levels has been observed after administration of S [318–510] -Fc, an analog of the portion of the Spike protein of the SARS-CoV virus family that binds the ACE2 (150). Similar conclusions have been reported in hyperoxic damage studies in animal models (mice). Hyperoxia significantly reduced the expression of pulmonary ACE2 and enzymatic activity, leading to an increase in Ang II and a reduction in Ang-(1-7) levels. In these experimental models, the administration of Diminazene Aceturate (DIZE), an ACE2 agonist, restored the levels of Ang-(1-7). On the other hand, the administration of the ACE2 inhibitor, MLN-4760 further worsened the reduction in Ang-(1-7) levels in line with the even more marked increase in Ang II (151). The tissues involved include all those that express ACE2 and in which it has been shown to have functionally relevant enzymatic activity such as the pulmonary epithelium, the renal and cardiovascular system and the CNS. With regard to CNS, we postulate that hyperactivation of the ACE/Ang II/AT1R axis may contribute to the onset of neuropsychiatric symptoms and on the cognitive sphere in two chronologically distinct steps: (1) in the course of infection by SARS-CoV-2 they would be a direct consequence of the increased stimulation of the AT1R receptor and of the hyperproduction of the Ang-(1-7) fragment and of the consequent reduced stimulation of the MasR; (2) in the medium-long term the effects on the CNS would be the consequence of two events: (a) neurotoxicity mediated by the ACE/Ang II/AT1R axis in the absence of the full neuroprotective effect of the ACE2/Ang-(1-7)/MasR axis; (b) from neurovascular damage mediated by cytokine storm syndrome, associated mainly with severe forms of COVID-19, which leads to an excessive immune response that damages blood vessels caused by an increase in proinflammatory cytokines such as IL-1, IL-6, and TNF-α (152).

Regarding the virus neurotropism and neurovirulence, SARS-CoV-2 can colonize and infect the CNS through two main pathogenetic modalities: (1) through a retrograde neurogenic pathway and (2) through fluids (hematogenous, lymphatic, and CSF pathway). In the first modality it colonizes the nerve endings of the eyes, of the nasal cavity, of the oropharynx and of the respiratory tract interacting with the ACE2 receptor expressed on the surface of the nerve endings themselves. Then, after the enzyme endocytosis process, it goes through a calmodulin-dependent retrograde calcium-transport pathway toward the brain nuclei. In the second modality SARS-CoV-2 penetrates the CNS due to damage of the BBB mediated by the cytokine storm and the virus reaches the CNS mainly via hematogenous and lymphatic route. This transition would also generate at the CNS level a reduced expression of ACE2 and a consequent functional imbalance between the ACE/Ang II/AT1R axis (hyperactivated) and the ACE2/Ang-(1-7)/MasR axis (hypo-activated). In support of the pathophysiological importance of this functional imbalance there is also epidemiological evidence that the mortality rate of elderly COVID-19 patients with high blood pressure, diabetes and cardiovascular pathologies that already have an ACE/Ang II/AT1R axis hyperactivation and a down-regulation of the ACE2/Ang-(1-7)/MasR axis, is higher than other patients with SARS-CoV-2 related infection (153). Furthermore, the male sex would be at greater risk in all age groups (154). Consistent with this epidemiological evidence, Xudong et al. showed a significant reduction in ACE2 expression in animal models during the aging process which was greater in rat males than in rat females (155).

We postulate that the clinical consequences on the CNS are also to be causally related to the decrease in the concentration of ACE2 and the consequent increase in the Ang II/Ang-(1-7) ratio with an imbalance between ACE/Ang II/AT1R axis and the ACE2/Ang-(1-7)/MasR axis. Specifically, this functional alteration of the RAAS cascade would account for both the neuropsychiatric comorbidities described in the short term and medium-long term cognitive impairment. Compared to the latter, the oxidative damage and neurotoxicity associated with hyperactivity of the Ang II on the AT1R receptors can lead to the onset of long-term cognitive damage.

In the first few months of COVID-19 spread, a controversial topic was the use of angiotensin receptor blocking drugs (ARB) and ACE inhibitors (ACEI) in patients with COVID-19. Given that previous studies reported a higher mortality rate in aged COVID-19 patients with comorbidities such as hypertension, and given that these patients are likely to be treated with ACEI or ARB, the concern was whether the use of ACEI and ARB could aggravate the related SARS-CoV-2 morbidity and mortality. Data from *in vivo* studies, on animal models of cardiovascular diseases, ACEIs, more than ARBs, have demonstrated the ability to determine the increase in ACE2 mRNA levels, thus being able to increase the expression of receptors used by SARS-CoV-2, thus facilitating the entry of the virus into the host. However, the change in protein levels is not always consistent with mRNA levels and sometimes also goes in the opposite direction. To date, it is still uncertain whether ACEIs and ARBs increase the protein expression of ACE2. According to Bian et al. (156), there is currently no clear, consistent and conclusive evidence indicating that ACEI and/or ARB increase the risk of SARS-CoV-2 infection, as well as injury to target organs. Consistently, so far it is not necessary to recommend discontinuation of ACEI/ARB for patients treated with hypertension. ARBs and ACEIs have also been shown to play a significant role in preserving cognitive functions. Indeed Ho et al. found that patients with hypertension had worse basal memory and executive function performance, as well as a faster decline in 3-year follow-up memory than patients with normal blood pressure values unless they were treated with ARBs (157). The study showed more preserved memory functions than patients treated with antihypertensive drugs belonging to other classes (157). Patients treated with ARB showed better performance times in memory functions than patients treated with other antihypertensive drugs (158, 159), and better learning memory performance over time compared to all other groups, including those with no high blood pressure and patients treated with antihypertensive drugs (157). These data suggest that ARB treatment is linked to higher memory retention level than other antihypertensive drugs, especially those that go through BBB.

Consistent with our hypothesis that COVID-19 patients are at a greater risk of developing or worsening cognitive decline, and considering the evidence that ARBs and ACEIs could be protective therapeutic tools against cognitive decline, at the time of writing there is no evidence to support the transition to other antihypertensive drugs but rather, treatment with antihypertensive drugs aimed at modulating the RAAS cascade could actually be a protective factor regarding the onset or worsening of cognitive impairment symptoms and signs.

In order to test our working hypothesis, our goal is to first complete an observational study to monitor cognitive functions in patients with COVID-19 who are accessible to neurocognitive testing. Then we aim to prospectively observe patients recovered from SARS-CoV-2 infection to follow the possible decline in cognitive functioning by relating it to the levels of activity of the RAAS cascade. Alongside this monitoring, our goal is to follow the evolutionary framework of neuroimaging to understand if there is a correlation between the decline of cognitive functions, instrumental signs of neurodegeneration and altered activity of the balance of the two arms of the RAAS cascade: ACE/Ang II/AT1R with proinflammatory activity and ACE2/Ang-(1-7)/MasR with anti-inflammatory properties. These data will then be cross-referenced with ACEI or ARB treatment to answer a still open question about the advisability of treatment with these antihypertensive drugs during SARS-CoV-2 infection also from a neuropsychiatric point of view.

## Conclusion

The SARS-CoV-2 pandemic represents an unprecedented challenge to healthcare systems around the world. At the onset of the pandemic, efforts by healthcare professionals and researchers focused on the urgency of treating patients who developed respiratory failure and needed assisted ventilation. However, it soon emerged that COVID-19 is a systemic pathology through the severe innate immune response and sustained rise of systemic cytokine levels (160). In fact, the innate immune response represents a predictor of mortality and severity of SARS-CoV-2 infection mediated through the production of cytokines and related inflammatory mediators found to be elevated such as interleukin-1β, interleukin-2, interleukin-2 receptor, interleukin- 4, interleukin-10, interleukin-18, interferon-γ, C-reactive protein, granulocyte colony-stimulating factor, interferon-γ, CXCL10, monocyte chemoattractant protein 1, macrophage inflammatory protein 1-α, and tumor necrosis factor-α and parallel reduction of T cell mediated response and reduction of lymphocyte count. Among the various organs involved in COVID-19 pathology is the CNS (160). This assumption is confirmed by numerous pieces of experimental evidence which have now definitively shown that SARS-CoV-2 has significant neurovirulence involving, as well as serious clinical pictures of interstitial pneumonia and consequent severe acute respiratory syndromes, neurological symptoms. The first evidence of this was the study of Mao et al. gathered in three designated special care centers for COVID-19 (Main District, West Branch, and Tumor Center) of the Union Hospital of Huazhong University of Science and Technology in Wuhan, China. Out of 214 hospitalized COVID-19 patients, more than a third had neurological symptoms (159). Patients with more severe forms of SARS-CoV-2 infection were more likely to develop neurological symptoms. In fact, according to Li, 89% of COVID-19 patients who need respiratory assistance in the Intensive Care Unit report neurological manifestations, the most common of which are headache, nausea, and vomiting (45).

In addition, a case of SARS-CoV-2 viral encephalitis was reported in Beijing's Ditan hospital on March 4, 2020 (161). This clinical case, together with the data that collected the SARS-CoV-2 RNA in the cerebrospinal fluid, would confirm the neurotropism and neuroinfectious potential of SARS-CoV-2. More recently, in a study by Varatharaj et al. in the United Kingdom, complications from SARS-CoV-2 were reported in a group of 125 patients with neurological involvement: of the 62% who presented with a cerebrovascular event, a rate of 74% had an ischemic stroke, 12% an intracerebral hemorrhage and 1% a CNS vasculitis. Twenty-three percent of COVID-19 patients had unspecified encephalopathy and 18% had encephalitis (127). The remaining 59% of COVID-19 patients had symptoms characterizing an altered mental state and met the diagnostic criteria for psychiatric diagnosis after evaluation by the consultant psychiatrist. Ninety-two percent of these diagnoses were of new onset. Specifically, 43% of patients had new-onset psychosis, 26% had a neurocognitive syndrome (similar to dementia) and finally, 17% had an affective disorder (127).

The neurological manifestations described seem to be currently supported by the following mechanisms, as previously described in agreement with Heneka et al. (19): (1) direct viral encephalitis, (2) systemic inflammation, (3) peripheral organ dysfunction (liver, kidney, lung), and (4) cerebrovascular changes. In most cases, however, neurological manifestations of COVID-19 may arise from a combination of the above.

We propose a fourth possible mechanism, linked to the pathogenesis of SARS-CoV-2 infection or to the binding of the virus to ACE2, consequent to the downregulation of this receptor and to the alteration of the dynamic balance between the two arms of the RAAS: (1) ACE/Ang II/AT1R with proinflammatory activity and (2) ACE2/Ang-(1-7)/MasR with anti-inflammatory properties. In this speculative article we have generated a hypothesis that we reserve the right to verify in clinical practice in the following months on patients with acute SARS-CoV-2 infection and in the follow-up in COVID-19 survivors.

## Data Availability Statement

The original contributions presented in the study are included in the article/supplementary materials, further inquiries can be directed to the corresponding author/s.

## Author Contributions

DD, FP, and AA contributed to the conception and design of this paper. FP wrote the first draft of the manuscript. MS and LC collaborated in the editing of the manuscript. LC elaborated the figures. All authors contributed to manuscript revision, read, and approved the submitted version.

## Conflict of Interest

The authors declare that the research was conducted in the absence of any commercial or financial relationships that could be construed as a potential conflict of interest.

## Footnotes

^1^ACE2 was first identified in 2000, as a homologous of a previously known protein, angiotensin-converting enzyme (ACE) (9, 10). Morphologically, it is a type I transmembrane protein which is comprised of 805 amino acids, a carboxy-terminal catalytic domain and alpha amino-terminal domain. In the catalytic domain an active site, called the zinc metalloprotein domain, matches 41.8% of the previously known ACE protein (10–12). Its corresponding gene is found in the short arm of the X chromosome (Xp22.2) (10). The ACE2 protein is found in many organs, specifically in the lungs, the kidneys, the testes, the intestine, the heart and, of particular interest for the aim of this paper, the brain (13).
